# A latent transition analysis of physical activity and screen-based sedentary behavior from adolescence to young adulthood

**DOI:** 10.1186/s12966-022-01339-4

**Published:** 2022-07-30

**Authors:** Kate Parker, Verity Cleland, Jim Dollman, Jacqui Della Gatta, Jennifer Hatt, Anna Timperio

**Affiliations:** 1 Institute for Physical Activity and Nutrition (IPAN), School of Exercise and Nutrition Sciences, Deakin University, Geelong, Australia; 2 Menzies Institute for Medical Research, University of Tasmania, Hobart, Tasmania Australia; 3 Alliance for Research in Exercise, Nutrition and Activity (ARENA), School of Health Sciences, University of South Australia, Adelaide, South Australia Australia

**Keywords:** Physical activity, Sedentary behavior, Transition, Typologies, Pathways, Youth

## Abstract

**Background:**

Distinct typologies of physical activity and screen-based sedentary behaviors are common during adolescence, but it is unknown how these change over time. This longitudinal study examined the stability of activity-related behavioral typologies over the transition out of secondary school.

**Methods:**

Year 11 students (penultimate school year) completed a self-report survey (baseline), which was repeated 2 years later (follow-up) (75% female, mean baseline age: 16.9 ± 0.4 years). Latent transition analysis identified typologies of physical activity and screen time behaviors and explored changes in typology membership between baseline and follow-up among those with complete data and who were not attending secondary school at follow-up (*n* = 803).

**Results:**

Three unique typologies were identified and labelled as: 1) Sedentary gamers (baseline: 17%; follow-up: 15%: high levels of screen behaviors, particularly video gaming); 2) Inactives (baseline: 46%; follow-up: 48%: low physical activities, average levels of screen behaviors); and 3) Actives (baseline: 37%; follow-up: 37%: high physical activities, low screen behaviors). Most participants remained in the same typology (83.2%), 8.5% transitioned to a typology with a more health-enhancing profile and 8.3% transitioned to a typology with a more detrimental behavioral profile.

**Conclusions:**

The high proportion within the ‘inactive’ typology and the stability of typologies over the transition period suggests that public health interventions are required to improve activity-related behavior typologies before adolescents leave secondary school.

**Supplementary Information:**

The online version contains supplementary material available at 10.1186/s12966-022-01339-4.

## Background

During the transition following secondary school education, young people are faced with expectations to become independent by gaining employment, enrolling in further study (e.g., tertiary education) and/or moving out of the family home. Evidence from longitudinal studies has demonstrated how our rapidly changing world is impacting on young people’s life patterns, particularly regarding an increased need for higher education and insecure employment conditions [1, 2]. Navigating this often complex and challenging transition period, and the loss of the structure of school life, may mean that physical activity becomes less of a priority and young people are exposed to more opportunities to engage in sedentary behavior.

A meta-analysis of 49 longitudinal studies showed evidence of modest declines in physical activity from adolescence to young adulthood, with a weighted mean difference (WMD) of − 5.2 minutes/day (95% CI: − 7.3 to − 3.1) over an average of approximately 3 years [3]. Additionally, a systematic review of 16 studies found evidence of low-to-moderate tracking in both the frequency and duration of physical activity during the transition period from adolescence to young adulthood [4]. To date, few studies have examined changes in, or tracking of, sedentary behavior over this transition period [4]. However, evidence from a meta-analysis of 130 studies found that children and adolescents (5–18 years) spend an average of 3.6 (range: 1.3–7.9) hours/day engaging in screen-based sedentary behavior [5], and data from a large-scale study of European adults reported a median sitting time of 5 hours/day in a large sample of 18–24 year-olds [6].

It is widely understood that physical activity provides health benefits, while engaging in excessive recreational screen time can be detrimental to health during adolescence [7, 8] and adulthood [9, 10]. This is particularly concerning given the aforementioned evidence that young people tend to engage in less physical activity and more sedentary behavior with increasing age, and that targeting an increase in physical activity does not necessarily result in reduced time spent in sedentary behavior [11]. Therefore, there is a need to examine physical activity and sedentary behavior (activity-related behaviors) simultaneously using a more holistic approach, rather than treating each behavior as distinct from one another. Using data-driven ‘clustering’ techniques, a growing body of research shows that distinct groups of adolescents can be identified based on shared patterns of behavior, termed typologies or profiles of behavior [11]. However, little research has examined changes in these typologies over time.

Emerging research has begun to use multi-level person-centered modelling techniques to explore whether changes in activity-related behaviors occur homogeneously among sub-groups of the population [12]. For example, Jago et al. explored latent transitions of device-measured physical activity and sedentary time typologies among young children aged 6–9 years [13]. Findings demonstrated considerable movement between typologies with the largest shifts seen among children transitioning towards typologies with less physical activity. Conversely, Dakin et al. explored latent transitions of eating behavior, physical activity and sedentary time typologies among adolescents with very little movement between typologies seen over 2 years [14]. However, to the authors’ knowledge, no studies have explored stability in typologies of physical activity and screen-based sedentary behaviors during the transition out of secondary school. Understanding how typologies change over this transition can inform efforts to ensure resilience to detrimental changes in behavior during a period of significant adaptation to new circumstances. This study therefore aimed to: 1) identify distinct latent typologies of activity-related behaviors during secondary school and 2 years later; 2) explore the stability of these typologies over the 2 years; and 3) explore differences in the stability of typologies according to whether participants engaged in further studies, had full-time employment, or had moved out of home 2 years later.

## Methods

Data were drawn from the first and last wave of ProjectADAPT, a longitudinal study of Year 11 students surveyed annually for 2 years. The study was approved by the Deakin University Human Research Ethics Advisory Group – Health (HEAG-H 159_2012) and relevant education authorities.

### Sample

Participants were recruited via schools (July 2013 to September 2014) or paid social media advertising (September–November 2014 and April–May 2015), as previously described [15]. A total of 232 public, Catholic and independent secondary schools in the state of Victoria, Australia with ≥50 Year 11 students (second last year of school) were approached to participate in the study, and 47 agreed. Participant information was distributed to Year 11 students and 411 completed consent forms were returned (response rate = 4.5%). To complement school recruitment, advertisements restricted to 16–17-year-olds from Victoria were placed on Facebook. Clicking on the advertisement directed students to a webpage with brief information about the study where individuals could register interest to receive further information and confirm eligibility (completing Year 11 and living in Victoria, Australia). There were 2770 registrations of interest, from which 665 completed consent forms were returned (response rate = 24%). The total number of consents received via these two approaches was 1076. Participants recruited in 2013 completed the surveys via telephone. Those recruited from 2014 to 15 could opt to complete all surveys via telephone or online. The baseline survey was completed by 1022 participants (76 by telephone) and the 2-year follow-up survey by 852 participants (39 by telephone), with a retention rate of 83%. Each participant maintained the same mode of data collection (telephone or online) through the study.

### Measures

Participants self-reported their age and gender at baseline, and their post-school circumstances/situation at the two-year follow-up, including whether or not they were engaged in further studies, in full-time employment, and their living arrangements (kind of residence they live in and who they live with compared to previous surveys, which was used to determine if they had moved out of home).

### Physical activity

The transport and leisure modules of the long-form, usual week version of the International Physical Activity Questionnaire (IPAQ) were repeated at baseline and follow-up [16]. Average total daily time spent engaging in active travel (by walking and cycling), leisure-time walking, leisure-time moderate-intensity physical activity (MPA), and leisure-time vigorous-intensity physical activity (VPA) were determined by multiplying the number of days on which they participated by duration per day and dividing by 7. Each variable was dichotomized according to whether participants recorded engaging in each activity for at least 10 minutes, due to the distribution of data for each of the four variables. Test-retest reliability was examined in a separate sample of Year 11 students (*n* = 82) who completed the survey approximately 2 weeks apart (mean 15.8 days); percent agreement between the two administrations ranged between 73 and 80% for each of these dichotomized variables (*n* = 81–82).

### Screen-based sedentary behavior

At baseline and follow-up, participants reported the usual amount of time (hours and/or minutes) that they spent sitting on weekdays and on weekend days to 1) watch TVs, DVDs or videos, 2) use a computer, laptop or tablet for entertainment, and 3) play electronic video games. For each behavior, durations on weekdays and weekends were summed and divided by seven to identify average total daily time spent engaging in each behavior for leisure purposes. Each of these items were then dichotomized as follows: leisure-time TV viewing, ≥120 mins/day vs < 120 mins/day according to the screen-time recommendations for youth [17]; leisure-time computer use, ≥60 mins/day vs < 60 mins/day based on previous research [18, 19]; and leisure-time video gaming, ‘no video gaming’ vs ‘video gaming’ as half of the sample did not engage in any video gaming. Test-retest reliability (percent agreement) of these three survey items dichotomized in the same way ranged between 79 to 86% (*n* = 78–81).

### Data analysis

The analytical sample included participants for whom activity-related behavior data were obtained at baseline and the two-year follow-up and indicated at follow-up that they were not attending secondary school. Differences in age, gender and time (mins/day) engaging in activity-related behaviors at baseline among the analytical sample and the remainder of the baseline sample (who did not complete the follow-up survey or were excluded based on secondary school attendance), and among those who completed the survey via telephone or online, were determined by chi-square tests and independent samples t-tests.

MPlus software (version 8) [20] was used to conduct latent transition analysis to identify activity-related behavior typologies during Year 11 and change or stability 2 years later (post-school). Only the dichotomized physical activity and screen-based sedentary behavior variables were used to identify the typologies. Latent transition analysis enables the identification of discrete subgroups (typologies) within a wider sample and their transitions over time. MPlus uses maximum likelihood estimation to handle missing data, thus allowing analyses to be conducted on the full sample (missing data for the activity-related behavior variables all ≤1%). Akaike Information Criteria (AIC) [21], Bayesian Information Criteria (BIC) [22], Entropy [23], class sizes (to ensure they were sufficient for subsequent analyses) and interpretability of typologies were compared to determine the optimal solution.

All further analyses were conducted in STATA (version 15.1) to describe each typology and transitions. One-way ANOVAs with Tukey post-hoc tests were used to compare time (mins/day) spent in each activity-related behavior between typologies at each of baseline and follow-up. Each activity-related behavior (mins/day) was compared between baseline and follow-up for the whole sample and for each typology using independent samples t-tests. Typology transitions were then classified based on participants’ transitions towards a more health-enhancing profile (improve), towards a detrimental behavioral profile (worsen), or remaining stable (stable) between the two time points. Differences between these transitions based on post-school situational pathways were then assessed using chi-square tests. The duration of each individual activity-related behavior was compared by transition (stable, improved or worsened) using one-way analysis of variance (ANOVA).

## Results

The final analytical sample included 803 participants (75% female, mean baseline age = 16.87 ± 0.43 years). No difference in age or gender was seen among those who did not complete follow-up (*n* = 170) or were excluded (*n* = 49) compared to the analytical sample, however there were significant differences in daily time spent engaging in all activity-related behaviors, except for TV viewing and computer use. Those in the analytical sample engaged in less active travel (24.28 ± 29.05 vs 31.07 ± 34.64 mins/day, *p* = 0.004), leisure time walking (11.17 ± 19.22 vs 16.75 ± 24.44 mins/day, *p* < 0.001), leisure time vigorous intensity physical activity (20.57 ± 30.49 vs 26.49 ± 33.03 mins/day, *p* = 0.012), leisure time moderate intensity physical activity (15.64 ± 22.75 vs 19.34 ± 26.96 mins/day, *p* = 0.040), and video gaming (14.56 ± 50.55 vs 23.20 ± 63.22 mins/day, *p* = 0.035) compared to those not in the final analytical sample. Those who completed the survey via telephone (mean = 77.71 ± 70.12 mins) differed in computer use only when compared to those completing the survey online (mean = 116.61 ± 106.57 mins, *p* = 0.030).

### Discrete latent classes at each time point

**Fig. 1:**
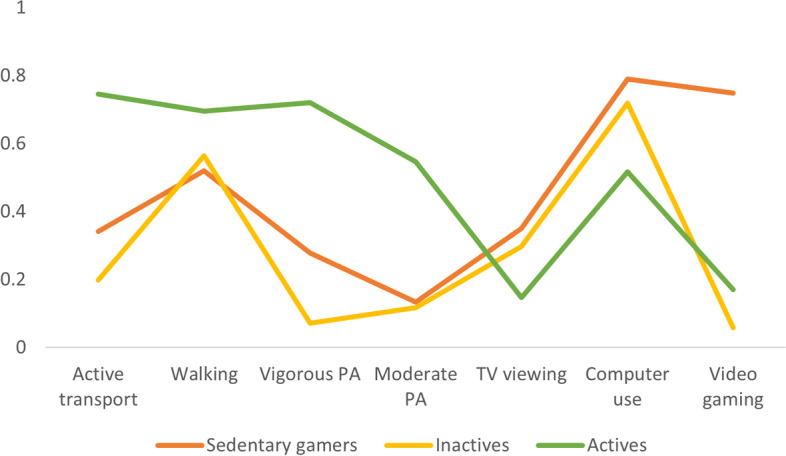
Baseline item-response probability plot indicating probability of typology membership according to each of the activity-related behaviors. PA: Physical Activity; Note that all physical activities and screen-based sedentary behaviors were performed during leisure time with the exception of active transport

**Fig. 2:**
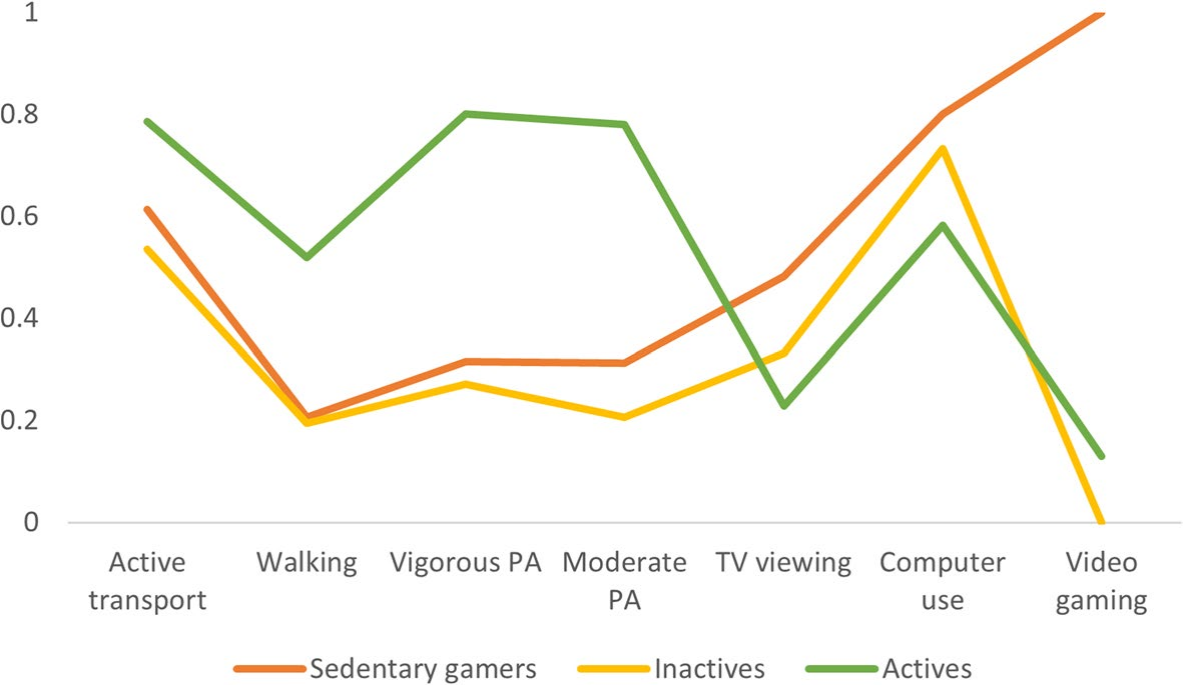
Two-year follow-up item-response probability plot indicating probability of typology membership according to each of the activity-related behaviors. PA: Physical Activity; Note that all physical activities and screen-based sedentary behaviors were performed during leisure time with the exception of active transport

The comparison of typology solutions at both time points, interpretation of statistical indicators (AIC, BIC and entropy), class sizes and overall interpretability revealed a three-typology solution as optimal (see supplementary Table 1). At both time points, these solutions yielded descriptively similar typologies, which were subsequently labelled as: 1) Sedentary gamers (baseline: 17%; follow-up: 15%); 2) Inactives (baseline: 46%; follow-up: 48%); and 3) Actives (baseline: 37%; follow-up: 37%). Compared to the other typologies, and at both time points, ‘Sedentary gamers’ were characterized by a high likelihood of engagement in excessive screen-based sedentary behaviors, particularly video gaming, ‘Inactives’ by a low probability of engagement in all physical activities and average likelihood of excessive screen-based sedentary behaviors, and ‘Actives’ by a high probability of engagement in all physical activities and lower likelihood of excessive screen-based sedentary behaviors (see Figs. 1 and 2, and Table 1). The ‘sedentary gamers’ typology was comprised of a greater proportion of males compared to the ‘Inactives’ and ‘Actives’ typologies at both baseline (56% compared to 15 and 22% respectively) and follow-up (59% compared to 16 and 22% respectively).

**Table 1:** Average daily duration (mins/day) in each activity-related behavior at baseline and follow-up according to typology membership

	Whole sample	Sedentary gamers	Inactives	Actives
	Mean ± S.D.	Mean ± S.D.	Mean ± S.D.	Mean ± S.D.
**Baseline (n)**	**803**	**129**	**376**	**298**
Active travel	24.28 ± 29.05	18.94 ± 23.34	21.89 ± 27.68	29.53 ± 32.01 ^*, #^
Leisure-time physical activity				
Walking	11.17 ± 19.22	7.26 ± 12.84	8.61 ± 15.50	16.10 ± 24.12 ^*, #^
Vigorous intensity	20.57 ± 30.49	13.26 ± 22.71	12.42 ± 19.71	34.04 ± 38.80 ^*, #^
Moderate intensity	15.64 ± 22.75	12.60 ± 21.71	8.82 ± 12.03	25.52 ± 29.17 ^*, #^
Leisure-time screen-based sedentary behavior				
TV viewing	97.50 ± 101.64	128.54 ± 131.22	100.51 ± 104.88 ^*^	79.43 ± 75.26 ^*, #^
Computer use	114.60 ± 105.07	165.59 ± 126.05	129.95 ± 106.19 ^*^	84.43 ± 83.14 ^*, #^
Video gaming	14.56 ± 50.55	57.46 ± 84.30	4.73 ± 34.37 ^*^	8.39 ± 36.83 ^#^
**Follow-up (n)**	**803**	**121**	**389**	**293**
Active travel	24.82 ± 34.97	18.71 ± 26.02	21.27 ± 31.66	31.91 ± 40.76 ^*, #^
Leisure-time physical activity				
Walking	10.35 ± 18.67	4.74 ± 10.52	6.68 ± 14.13	17.41 ± 23.63 ^*, #^
Vigorous intensity	*17.36 ± 26.43*	13.34 ± 21.98	10.21 ± 18.52	28.66 ± 32.58 ^*, #^
Moderate intensity	*12.44 ± 18.74*	10.40 ± 19.55	6.90 ± 12.17	21.00 ± 23.16 ^*, #^
Leisure-time screen-based sedentary behavior				
TV viewing	100.46 ± 95.79	145.20 ± 134.42	103.53 ± 94.06 ^*^	77.71 ± 67.58 ^*, #^
Computer use	123.44 ± 118.54	172.22 ± 153.12	134.36 ± 123.16 ^*^	89.96 ± 86.31 ^*, #^
Video gaming	15.03 ± 53.36	83.59 ± 103.59	0.0 ± 0.0 ^*^	6.74 ± 33.71 ^#^

Italicized and underlined follow-up value indicates significant difference between time points based on independent t-tests (*p* < 0.05);

Comparisons between typologies at each time point: ^*^p < 0.05, compared with adolescents in the ‘sedentary gamers’ typology based on ANOVA; ^#^*p* < 0.05, compared with adolescents in the ‘inactives’ typology based on ANOVA

### Typology transitions from baseline to follow-up

**Fig. 3:**
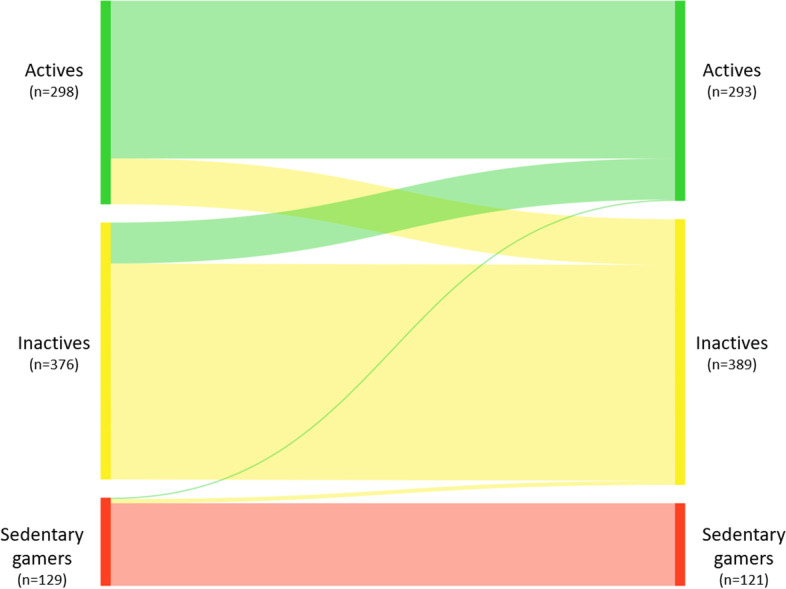
Transitions between typologies from baseline to two-year follow-up

Table 2 and Fig. 3 provide an overview of the nine potential typology transitions from the latent transition analysis, overall and according to baseline typology. In brief, most adolescents remained stable in their typology membership during the transition into young adulthood (83.2%). Overall, just 8.5% of participants transitioned towards more health-enhancing activity-related behavior typology (improved), and 8.3% transitioned towards an activity-related behavior typology that is considered more detrimental for health (worsened). Very few participants transitioned out of the ‘sedentary gamers’ typology, and no participants transitioned into this typology.

**Table 2:** Typology transitions

Typology at baseline	Typology at follow-up	n	Overall transition (%)	Transition within baseline typology (%)
Sedentary gamers (*n* = 129)	Actives	2	0.2	1.6
	Sedentary gamers (stable)	121	15.1	93.8
	Inactives	6	0.7	4.6
Inactives (*n* = 376)	Actives	60	7.5	16.0
	Sedentary gamers	0	0.0	0.0
	Inactives (stable)	316	39.4	84.0
Actives (*n* = 298)	Actives (stable)	231	28.8	77.5
	Sedentary gamers	0	0.0	0.0
	Inactives	67	8.3	22.5

### Characterizing the typology transitions (Table 3)

Average duration (mins/day) of walking, VPA, and MPA during leisure time differed significantly between all three typology transitions. No differences between the typology transitions were seen for TV viewing, computer use or video gaming. There were no significant differences between the typology transitions according to whether participants were engaged in further studies, full-time employment, or had moved out of home at follow-up.

**Table 3:** Characteristics of the typology transitions

	n	Transitioned to more active/less sedentary typology (***n*** = 68)	Stable (***n*** = 668)	Transitioned to less active/more sedentary typology (***n*** = 67)
Change in daily minutes (follow-up – baseline; mean ± S.D.)				
Active travel	798	11.11 ± 41.42	1.25 ± 36.74	− 17.06 ± 39.82 ^*, #^
Leisure-time physical activity				
Walking	793	12.16 ± 30.25	−1.22 ± 21.63 ^*^	−8.75 ± 20.74 ^*, #^
Vigorous intensity	797	17.97 ± 24.93	− 3.89 ± 31.49 ^*^	−17.90 ± 25.53 ^*, #^
Moderate intensity	798	16.58 ± 24.22	−4.16 ± 25.43 ^*^	−13.38 ± 10.76 ^*, #^
Leisure-time screen-based sedentary behavior				
TV viewing	790	−10.55 ± 99.87	5.30 ± 125.09	−6.68 ± 101.83
Computer use	788	−22.11 ± 116.12	12.17 ± 129.60	6.63 ± 135.47
Video gaming	798	−2.47 ± 22.53	1.99 ± 56.03	−11.62 ± 35.36
Follow-up situational pathways (% yes)				
Further study	796	82.1	78.6	83.3
Full-time employment	800	13.4	7.8	8.9
Moved out of home	803	14.7	20.5	26.9

^*^p < 0.05, compared with adolescents who transitioned to a more active/less sedentary typology; ^#^*p* < 0.05, compared with adolescents who remained stable in their typology membership

## Discussion

This study provides a novel exploration of the nature of clustered activity-related behaviors in late adolescence and how these change over the transition out of secondary school. Within this sample, three unique activity-related behavior typologies were identified during both baseline and follow-up. Each of the three typologies had > 75% stability suggesting that there was little change in how the activities clustered (i.e., patterns of behavior) over the 2 years among this sample. Considering this, and that the ‘inactive’ typology was the most common of the three typologies at both time points, public health interventions may be most important while adolescents are still at school, prior to Year 11, to improve the combined range of activity-related behaviors during school. To date, there is limited evidence of effectiveness of interventions to increase physical activity or reduce sedentary behaviors amongst adolescents, particularly outside of school hours [24, 25], warranting further research and investment. The current findings additionally suggest that strategies should be tailored according to activity-related behavior patterns.

Transitions between typologies appeared to be mainly driven by changes in physical activity, and not changes in screen-time sedentary behavior. These shifts are useful to understand when determining which behaviors should be targeted in interventions to help adolescents to avoid adoption of detrimental behavioral patterns after leaving school. However, to strengthen the development of tailored strategies, future research should additionally explore the modifiable factors influencing typology transitions.

Overall, despite the transition out of secondary school being considered one of great change that can disrupt behavior patterns as individuals adapt to new circumstances [1, 2], individuals generally remained in the same behavioral typology and typology transitions did not appear to differ according to post-school pathway or circumstance. Additional analyses (data not shown) also showed no differences in all possible typology transitions according to situational pathways. Cross-sectionally, past studies have found differences in individual types of behaviors according to post-school situation. For example, university students spend more time sitting than the general population of young adults and young adults who live in the family home or their own place are more physically active than those who live on a campus [6, 26, 27]. Given the mix of behaviors participants engaged in (typologies) remained stable for over 80%, there was little change on average in most behaviors among those who remained in the same typology, and post-school pathways did not appear to impact these typologies, this study indicates that behavioral patterns (defined as a mix of behaviors) may become ingrained during secondary school. It is possible, however, that behavioral typology transitions may differ by other situational changes or pathways following secondary school that were not considered here, such as job type, rather than employment as a whole. Several studies have shown differences in physical activity and sedentary behavior across different occupations or occupational categories [6, 28].

This study is among the first to use a novel person-centered statistical technique to explore latent transitions in typologies of movement behaviors over the transition period out of secondary school. This is both a strength and limitation. The use of a person-centered statistical approach means that results are not directly transferrable to other samples of youth as the typology solutions are data dependent. Therefore, this study should be replicated in other samples of school leavers. However, the approach allows for identification of sub-groups of the population that are unique based on patterning of different activity-related behaviours that would otherwise be undetected using more traditional, variable-centered approaches. The study is also unique in examining the immediate transition out of secondary school; however, it is possible that insufficient time had elapsed for changes in behavioral typologies to occur following school. The lack of change in typology membership could also be explained through the ‘carry-over hypothesis’ suggesting that physical activity behaviors during younger years carry over to adulthood, or the ‘habit formation hypothesis’ which suggests that participation in physical activity becomes automatic once it has been repeatedly practiced over time [29].

While retention of the sample was high, the response rate from recruitment via schools was low (4.5%) and the sample comprised of a much higher proportion of girls than in the population [30]. Additionally, the proportions of the total sample who were engaged in further study (79%) and full-time employment (75%) were also higher than the general Australian population of the same age group [31, 32]. There is established evidence regarding an association between employment and education with activity-related behaviors amongst young adults [6, 33], therefore potential selection bias is another limitation that must be taken into consideration when interpreting the study findings. The reliance on self-report and the two modes of completion (online or telephone) were further limitations that may have also introduced some bias. However, the IPAQ is an internationally recognized valid and reliable questionnaire [16] commonly used to assess physical activity and screen-based sedentary behavior in large studies, and each participant used the same mode of completion for each survey. The physical activity variables were dichotomized at 10 mins/day. It is possible that different results may have been found if a higher value had been chosen. However, the variety of behaviors (i.e., multiple sedentary behavior indicators) allows for a deeper insight into the specific movement behaviors that may be most in need of targeting through public health interventions to improve overall health of young people prior to the transition period following secondary school. Future studies should consider utilizing a combination of subjective and objective measures of movement behaviors to increase validity, while not losing valuable insight into the individual behaviors that comprise potential typologies.

## Conclusions

Very few adolescents transitioned towards more health-enhancing or detrimental profiles of activity-related behaviors during the period following secondary school, and no differences were seen by post-school situational pathways. As most young people in this study remained in a typology defined by high engagement in screen-based sedentary behaviors or inactivity after leaving secondary school, the findings from this study suggest that public health interventions need to target adolescents before they transition out of secondary school. Future studies should explore whether activity-related behavior typologies remain stable over a longer follow-up period.

## Supplementary Information

**Additional file 1: Supplementary Table 1.** Interpretation of statistical indicators for models with 2–6 classes (latent transition analysis)

**Additional file 2.** STROBE Statement—checklist of items that should be included in reports of observational studies.

## Data Availability

The datasets analyzed during the current study are not publicly available but are available from the corresponding author on reasonable request.

## Abbreviations

AIC: Akaike Information Criteria
ANOVA: Analysis of variance
BIC: Bayesian Information Criteria
IPAQ: International Physical Activity Questionnaire
MPA: Moderate-intensity physical activity
VPA: Vigorous-intensity physical activity
