# Supplementary material for: A latent transition analysis of physical activity and screen-based sedentary behavior from adolescence to young adulthood
Source: Int J Behav Nutr Phys Act. 2022 Jul 30;19:98. doi: 10.1186/s12966-022-01339-4 (PMC9338621; doi:10.1186/s12966-022-01339-4)
Supplement: Supplementary file 1 — Additional file 1: Supplementary Table 1. Interpretation of statistical indicators for models with 2–6 classes (latent transition analysis) [file 12966_2022_1339_MOESM1_ESM.docx]

Supplementary table 1. Interpretation of statistical indicators for models with 2-6 classes (latent transition analysis)

|  | **2 classes** | **3 classes** | **4 classes** | **5 classes** | **6 classes** |
| --- | --- | --- | --- | --- | --- |
| **AIC** | 13500.183 | 13381.069 | 13269.674 | 13217.207 | 13278.016 |
| **BIC** | 13645.522 | 13615.487 | 13799.458 | 14183.008 | 14820.485 |
| **Entropy** | 0.593 | 0.738 | 0.713 | 0.806 | 0.852 |
| **Class size**  **(baseline)** | 1 = 450  2 = 353 | 1 = 387  2 = 108  3 = 308 | 1 = 214  2 = 111  3 = 87  4 = 391 | 1 = 118  2 = 43  3 = 80  4 = 308  5 = 254 | 1 = 137  2 = 21  3 = 41  4 = 175  5 = 138  6 = 291 |
| **Class size**  **(follow-up)** | 1 = 439  2 = 364 | 1 = 318  2 = 379  3 = 106 | 1 = 200  2 = 143  3 = 180  4 = 280 | 1 = 91  2 = 55  3 = 184  4 = 206  5 = 267 | 1 = 65  2 = 76  3 = 80  4 = 235  5 = 199  6 = 148 |
